# Evaluation of deep learning tools in medical diagnosis and treatment of cancer: research analysis of clinical and randomized clinical trials

**DOI:** 10.3389/fnetp.2025.1578562

**Published:** 2026-01-05

**Authors:** Rawad Hodeify

**Affiliations:** Department of Biotechnology, School of Arts and Sciences, American University of Ras Al Khaimah, Ras Al Khaimah, United Arab Emirates

**Keywords:** algorithm, cancer, cancer diagnosis, classification, clinical trials, deep learning, neural networks, prediction

## Abstract

Artificial Intelligence and machine learning tools have brought a revolution in the healthcare sector. This has allowed healthcare providers, patients, and public to be at pole position -amidst the key consideration and barriers-to attain precision and personalized medicine. Deep Learning (DL) is a branch of machine learning and AI that has become transformative for healthcare and biomedicine, providing the ability to analyze large, complicated data, capture abstract patterns, and present fast and accurate predictions. DL models are based on complex neural networks that emulate biological neural networks. In this paper, our goal is to evaluate DL algorithms in clinical trials stratified per cancer type and present future perspectives on the most promising DL approaches. We systematically reviewed articles on deep learning in cancer diagnostics in studies published in the Pubmed database. The searched literature included two types of articles, clinical trials, and randomized controlled trials. The deep learning algorithms used in the targeted literature are reviewed, and then we evaluated the performance of the algorithms used in disease prediction and prognosis. We aim to highlight the promising DL approaches reported per cancer type. Finally, we present current limitations and potential recommendations in large-scale implementation of deep learning and AI in cancer care.

## 1 Introduction

With the emergence of artificial intelligence (AI) and its development during the past years, the conventional medical diagnosis and patient treatment have advanced with higher accuracy and lower human workload. The integration of AI into medical diagnosis, across different specialties, greatly minimized the time required for diagnosis and significantly improved the diagnosis accuracy and efficiency (Liu et al., 2021). In addition, over the past decade, AI has proved to be instrumental in improving healthcare and medicine in terms of medical imaging, diagnosis and personalized treatment, drug discovery and development, monitoring and tracking chronic diseases, predictive analysis and clinical decision support by providing real-time information and alerts based on patient data (Jiang et al., 2020). Furthermore, AI has been used to analyze large amounts of data to identify patterns and gain new insights, thus boosting medical and biomedical research (Vodanović et al., 2023). In nearly all diseases, radiology is the innate basis of the diagnostic process. Numerous applications of AI in radiology have emerged during the last couple of years. AI deep learning (DL) algorithms have proven, in certain cases of MRI data analysis, to be superior to human observation (Liu et al., 2021). The progression of computer vision DL algorithms has increased interest in applying AI and machine learning (ML) to tumor pathology. The use of AI in pathology has developed from expert systems to ML and then to DL. With DL from raw data instead of rules and features based on expert definitions or expertise, it has proven to be easier to conduct and resulting in higher accuracy and precision in decisions or predictions made (Vodanović et al., 2023). Such applications of DL in medicine have helped in overcoming subjective visual assessment and integrating precision in tumor treatment (Jiang et al., 2020). In addition, DL has proved efficient in classifying tumor grades and distinguishing low to medium risk cases. With certain DL-based models achieving an accuracy of 91% in differentiating between normal tissue, low-grade and high-grade adenocarcinoma tissue, DL proves to be capable in generating objective and consistent results in classifying tumor grading (Jiang et al., 2020). Another specialty area in medicine that has benefited from the capabilities and potential of AI and DL-based models is dentistry. Using data from medical images (CT scans and X-rays), AI can provide superior help in identifying and diagnosing conditions, such as cavities and periodontal disease, and provide personalized treatment plans. AI has also proved to be a great support in diagnosing oral pathology offering great potential in detecting tumor tissues in provided samples (Vodanović et al., 2023). It is evident that DL has a great potential and impact on healthcare and support to the diagnosis, detection, personalized treatment and predictive analysis of several diseases. In this work we evaluated the performance of DL applications in cancer care, included in clinical and randomized clinical trials. Our aim is to reach a strong evaluation on the beneficial aspects of DL in cancer by summarizing the type of models, input data, and the level of performance of these models. Finally, we categorized these studies into cancer types, then we evaluated the performance of the various DL models, to be able to arrive at helpful interpretations.

## 2 Relevant literature of DL models in several cancer types

The following section will provide an overview of the selected studies that were conducted using DL by cancer type. The reviewed literature included studies implementing DL models in 13 types of cancer (Table 1) from 22 studies [Supplementary File 1], including breast cancer (Yin et al., 2022; Zheng et al., 2024; Ma et al., 2023; Yu et al., 2021; Meng et al., 2022), thyroid cancer (Zhan et al., 2024), brain cancer (Jayachandran Preetha et al., 2021), gastric atrophy (Fang et al., 2024), hepatic cancer (Wu et al., 2022; Liu et al., 2023; Chen et al., 2022), prostate cancer (Pellicer-Valero et al., 2022; Zhang et al., 2024; Johnsson et al., 2022), endometrial cancer (Fremond et al., 2023), renal clear cell carcinoma (Wen-Zhi et al., 2022), pancreatic cancer (Chen et al., 2023; Zhang et al., 2021), oropharyngeal carcinoma (Kann et al., 2023), lung cancer (Zhang et al., 2022), esophageal adenocarcinoma (He et al., 2016), and leukemia (Arabyarmohammadi et al., 2022).

**TABLE 1:** Description of studies, type of samples, patient size, and type of DL algorithms used.

Disease type	Samples	Patient number	Algorithm
Breast Cancer (Yin et al., 2022)	MRI	136	CNN
Breast Cancer (Zheng et al., 2024)	MRI	341	CNN
Breast Cancer (Ma et al., 2023)	CT	230	High Resolution Net
Breast Cancer (Yu et al., 2021)	US images	3623	CNN
Breast Cancer (Meng et al., 2022)	MRI	310	DTL - CNN
Thyroid (Zhan et al., 2024)	US images	500	CNN
Brain (Jayachandran Preetha et al., 2021)	MRI	19	CNN
Intestinal Metaplasia (Fang et al., 2024)	Histology images	545	Semi-supervised DNN
Liver (Wu et al., 2022)	US images	513	CNN
Liver (Liu et al., 2023)	CT	151	RCNN
Liver (Chen et al., 2022)	CT	595	Deep Learning Network
Prostate (Pellicer-Valero et al., 2022)	MRI	490	CNN
Prostate (Zhang et al., 2024)	MRI	211	DTL - Classification model
Prostate (Johnsson et al., 2022)	CT	339	aPROMISE Software
Endometrial (Fremond et al., 2023)	H&E slide images	2028	DTL - Classification model
Renal Clear Cell Carcinoma (Wen-Zhi et al., 2022)	Tabulated data	878	Multivariate Prediction Model
Pancreatic (Chen et al., 2023)	CT	546	CNN
Pancreatic (Zhang et al., 2021)	CT	98	DTL - CNN
Oropharyngeal carcinoma (Kann et al., 2023)	CT	360	(3D) Convolutional Neural Network
Lung (Zhang et al., 2022)	CT	720	CNN
Esophageal adenocarcinoma (He et al., 2016)	Fluorescence endoscopy images	31	CNN
Leukaemia (Arabyarmohammadi et al., 2022)	Aspirate slide images	92	DTL - Classification model

### 2.1 Breast cancer

A significant part of literature is in the context of breast cancer, providing an advantage to support the overall recommendations of using DL in this specific type of cancer. A study by Yin et al. (2022) in China included 136 patients with breast cancer in their study. All 136 patients were pathologically positive for invasive breast cancers. Furthermore, immunohistochemical analysis of samples from patients allowed identification of molecular subtypes of cancer. The input data used for building CNN deep learning model was magnetic resonance images (MRIs) taken from patients in prone position. Multiple CNN models were created based on the MRI-imaging models. The developed models demonstrated different predictive performance levels for each molecular subtype. One CNN model showed moderate performance levels in prediction of four molecular subtypes with Area Under the Curve (AUC) between 0.639 and 0.697. Another CNN model achieved better results (AUC 0.851) in separation of the triple negative subtype from all other subtypes. A third CNN model showed excellent performance in separating triple-negative and HER2‐enriched types from all other types (AUCs, 0.920 and 0.885) and positive predictive value PPV (0.889 and 0.936). The findings in this study demonstrated the capability of CNN architectures to discriminate between the four molecular subtypes of breast cancer, with variable accuracy. Importantly, some of the developed models showed excellent performance in differentiating specific molecular subtypes such as triple negative and HER2-enriched subtypes from the rest. In another study by Zheng et al. (2024), an MRI-based CNN (PCMM‐Net) model was developed including clinico‐radiological features as image inputs. The aim of the study was to develop a model to differentiate lymphovascular invasion (LVI) from regional and systemic lymph node metastases, given its important implications in managing treatment against breast cancer. The study included 341 females with confirmed invasive breast cancer who underwent pretreatment contrast‐enhanced MRI. The PCMM-Net architecture exhibited promising performance in LVI prediction in breast cancer patients, achieving excellent performance metrics (AUC of 0.843; accuracy of 0.824, sensitivity of 0.818, and specificity of 0.816). The PCMM-Net architecture surpassed other machine learning models using the clinico‐radiological features, model-based multimodal network (MM‐Net), and radiomics model. The supremacy of the developed model is attributed to the ability of the deep learning model to automatically extract intrinsic features associated with LVI, eliminating the need for manual input of features used in conventional radiomics. In a study by Ma et al. (2023), deep learning tools were used to predict radiation dose in patients with invasive breast cancer. In this study, CT scans from 208 patients from four nationwide centers in China were used to develop the DL model, and from an additional 22 patients from another three centers serving as the external testing cohort. The high-resolution-based DL was developed to detect the lead wire marker in each slice of the CT images automatically. The developed model allows prediction of the lung dose based on the related organ features with achieved precision and recall of 0.860 and 0.883, respectively, in the internal testing cohort, and reached 0.848 and 0.830, respectively, in the external testing cohort. This demonstrates the stable performance of the presented model which can highly be useful in selecting more appropriate irradiation procedures for individual patients. This can be further investigated in radiation therapy for other organs. In Yu et al. (2021) study, convolutional neural network (CNN) models were built to distinguish between benign and malignant tumors of the breast, as well as to identify masses and adenosis. Breast images were collected using different modalities of 2D ultrasound images. The DL model was designed to detect location of the mass and to classify the benign from tumor masses. The detection module included feature extraction using ResNet50. The CNN model included 15,648 images from a total of 3623 patients, divided almost equally into training and testing sets. The CNN model showed better performance and faster classification compared to results by ultrasonologists. The accuracy achieved by the CNN model was 0.89 and CPU processing time was 1s, while the accuracy of the ultrasonologist classification was 0.45 and the shortest time 83s. Finally, Meng et al. (2022) used deep learning to differentiate malignant from benign tumors using dynamic contrast-enhanced magnetic resonance breast images. In this study, 4260 and 4140 images of benign and malignant lesions were collected respectively from 310 patients. The data was split into training and testing at ratio 9:1, unlike the common ratio of 7:3. The model was established and tuned using four strategies. The best achieved accuracy was 0.75 and AUC/ROC of 0.79.

### 2.2 Thyroid cancer

In thyroid cancer, Zhan et al. (2024) utilized deep learning techniques to classify thyroid nodules as benign or malignant, based on ultrasound images. A total of 500 patients with detected thyroid nodules were split into training and testing sets at a ratio (70:30), respectively. The results showed that the deep learning model showed better prediction of benign and malignant thyroid nodules with an AUC of 0.870, compared to traditional methods such as Thyroid Imaging Reporting and Data System classification with obtained AUC of 0.761.

### 2.3 Brain cancer

In brain cancer, a study by Jayachandran Preetha et al. (2021) used deep learning architectures to study the diagnostic performance of post-contrast MRI, to potentially reduce the necessity of gadolinium-based contrast agents administration during MRI scans. This study included 2000 patients from over 200 institutions in Germany. The established deep convolutional neural networks (dCNN) achieved structural similarity index measure of 0.818 for predicting contrast enhancement on synthetic post-contrast T1-weighted MRI. The synthetic post-contrast T1-weighted MRI allows assessment of the patient’s response to treatment with similar performance to true post-contrast T1-weighted images.

### 2.4 Gastric cancer

In gastric cancer, Fang et al. (2024) introduced a deep learning algorithm using histology slides images from a total of 546 patients from 13 Chinese tertiary hospitals. The patients were split into a training set (349 patients with 1745 images), testing set (109 patients with 545 images), and a validation set (87 patients with 435 images). Interestingly, the performance of a deep learning model to diagnose inflammation, activity, atrophy, and intestinal metaplasia in gastric biopsy specimens was better than those of 10 pathologists.

### 2.5 Hepatic cancer

In hepatic cancer, Wu et al. (2022) study included a total of 513 patients with hepatocellular carcinoma (HCC) who underwent preoperative gray-scale-ultrasonic imaging. In this study, a deep CNN ResNet 18 model was developed to analyze ultrasonic images of HCC and peritumoral images to develop a prognostic and differentiation framework. The model showed good performance in prediction of early recurrence (validation index, C-index 0.695), late recurrence (C-index 0.715), and recurrence-free survival (C-index 0.721) in the validation cohort, which was superior to the clinical model and ultrasonic semantic model. The study by Liu et al. (2023) developed a Faster region-based convolutional neural networks (RCNN) using computed tomographic (CT) scans from 109 patients with common hepatocellular carcinoma (CHCC) and 42 patients with primary clear cell carcinoma of the liver (PCCCL). The model recorded superior performance with a recall of 0.951 and 0.960 for diagnosing PCCCL and CHCC, respectively. The model accuracy for accurately diagnosing PCCCL and CHCC was 0.962. Interestingly, the results of the model were significantly better than those of the two doctors. The average time required for the model to diagnose each case was 4 s compared to 120 s and 50 s for the two doctors.

In an interesting study, Chen et al. (2022) compared 6 models, including a deep learning model, for predicting short-term mortality in HCC patients with lung metastasis using demographic and clinicopathological (tabulated) data. The selected variables were chemotherapy, age, radiation, tumor size, grade, surgery, and stage of the tumor. Although the deep learning model achieved acceptable performance, it fell behind Random Forest and Logistic Regression, suggesting that DL models can have supremacy with imaging data.

### 2.6 Prostate cancer

In prostate cancer, Pellicer-Valero et al. (2022) used Retina U-Net27 CNN architecture, which allows for the simultaneous detection, segmentation, and classification of prostate cancer lesions, using multi-parametric magnetic resonance imaging (mpMRI). The model was used on 490 mpMRIs (one per patient) for training/validation and 75 for testing from two different datasets. In the test set, it achieves an excellent classification of lesion-level in the two datasets. The sensitivity and specificity of the CNN model was superior to those of the radiologists. Zhang et al. (2024) utilized ResNet50 as the model for extracting deep learning features from MR images in a retrospective study with 454 prostate cancer patients in China. The deep learning extracted features were combined with radiomics and pathomics data to create predictive models. The results showed that the best prediction model (SVM) is the one that combined radiomics features, deep learning features, and pathomics features using the SVM model, with an AUC value of 0.93.

### 2.7 Endometrial cancer

Fremond et al. (2023) developed a deep learning pipeline(im4MEC) using haematoxylin and eosin-stained whole-slide images of endometrial cancer from 2028 patients. The im4MEC model achieved AUC/ROC of 0.876 on the independent test set with class wise AUC/ROCs between (0.844–0.928) on the four molecular classes of endometrial cancer. This study demonstrated the potential advantage of deep learning tools to allow molecular classification and disease management of endometrial cancer by using standard diagnostic histology sections.

### 2.8 Renal cancer

In renal clear cell carcinoma, Wen-Zhi et al. (2022) established a deep learning CNN model to predict pathological staging of renal clear cell carcinoma using laboratory results and symptoms. The total number of patients was 878 split into test set (n = 702) or the verification set (n = 176). The CNN models showed good performance in predicting T-staging and of renal clear cell carcinoma with AUC 0.933-0.947 with lower prediction of G-grading. The study suggested that CNN based models with laboratory data can achieve good performance in renal clear cell carcinoma staging and decrease the need for invasive tests and reduce the financial burden.

### 2.9 Pancreatic cancer

In pancreatic cancer, a study by Chen et al. (2023) using contrast-enhanced CT scans obtained from 546 patients and 733 healthy subjects achieved similar sensitivity (89.9%) in the internal test set to that of the radiologists (96.1% sensitivity). The results were validated using 1473^−ΔΔCT^ scans from external test sets from Taiwan. In a paper published in 2021, Zhang et al. (2021) highlighted the shortcomings of several radiomics studies that aim to provide diagnostic and prognostic patient information directly from images. As most of these studies lack testing control, some of the output features may be the result of type I errors (Kann et al., 2023; Zhang et al., 2022). In their study, the authors proposed extraction of features through transfer learning using a CNN model (LungTrans) trained previously on non-small cell lung cancer (NSCLC) CT images (He et al., 2016). The included data for analysis was from two cohorts from two independent hospitals in Canada. The authors compared the performance of a risk score-based method which fuses deep transfer learning and radiomics features with four existing feature reduction methods. The AUC for the existing methods ranges from 0.5 to 0.6 on the test set of 30 patients, while the proposed method fusing deep transfer learning and radiomics features achieved the highest AUC of 0.84.

### 2.10 Oropharyngeal carcinoma

In a study on human papillomavirus (HPV)-associated oropharyngeal carcinoma, Kann et al. (2023) utilized deep learning algorithms for extranodal extension (ENE) prediction. The model was developed using 178 collected CT scans from ECOG-ACRIN Cancer Research Group multicenter trial E3311 in the US, and the performance was compared with four board-certified head and neck radiologists. The deep learning algorithm performance achieved an AUC of 0.857 which surpassed those of the four readers (0.66, 0.71, 0.70, 0.63). The authors pointed out the importance of considering the tradeoff between sensitivity and specificity of ENE identification. Importantly, they concluded that adapting this promising framework in a clinical setting will require clinician opinion to support optimal decision-making and reduce patient risk.

### 2.11 Lung cancer

In a study by Zhang et al. (2022), CNN and Random Forest (RF) models were developed using thoracic CT scans to predict benign and malignant solid pulmonary nodules. The study retrospectively enrolled a total of 720 patients, with 348 benign and 372 malignant cases. Thoracic CT scans images acquired before treatment and clinical features of each patient were used as input data. The dataset was split into training and testing with ratio 70%–30%. Clinical features included age, gender, smoking status, personal and family history of malignancy, nodule size and location, pathology, and clinical stage. CNN and RF based models with CT scans, clinical features, or both were compared. The performance levels for CNN models with and without clinical features demonstrated better diagnostic value in comparison to RF models. The CNN model with clinical features achieved an accuracy of 0.783, an AUC score of 0.819, and sensitivity of 0.778. The RF models with combined features achieved an accuracy of 0.704, AUC of 0.811, and sensitivity of 0.616. Interestingly, the CNN model with clinical features exhibited better AUC, accuracy, and specificity compared to the metrics achieved by two radiologists. Furthermore, the sensitivity of the CNN model was equivalent to that of the first radiologist but lower than that of the second radiologist. Although the established CNN models achieved promising performance, the study lacked external validation.

## 3 Data collection and analysis procedures

Deep learning has shown to be useful due to its capability to discover hidden patterns in raw data that is often complex and volumetric. The focus on cancer stems from the complexity of the disease leading to escalated complexity in the associated data. In this paper we aim to analyze published work on DL and cancer and present future insights about use of DL in cancer diagnosis and therapy.

**FIGURE 1:**
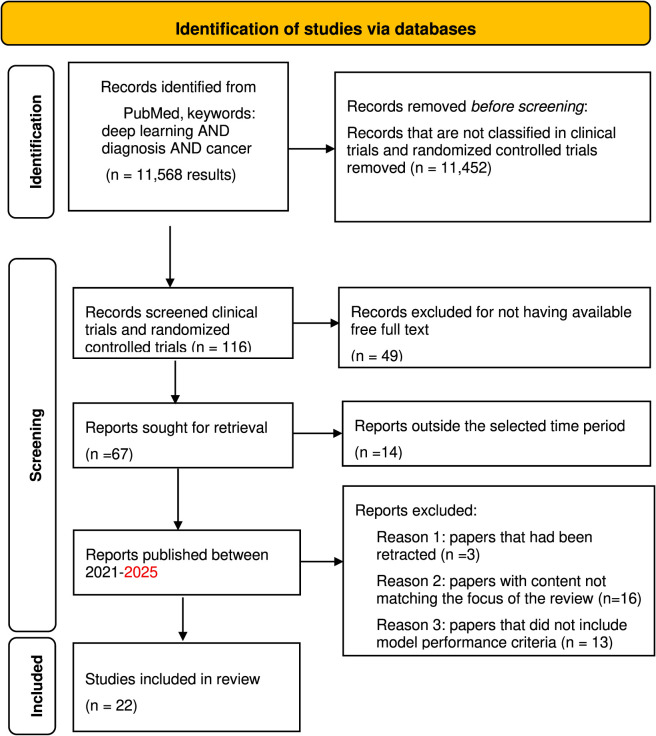
PRISMA flowchart.

We followed the preferred reporting items for systematic reviews and meta-analyses (PRISMA) criteria (Moher et al., 2008; Moosapour et al., 2021). Our selection of papers included in the analysis followed two stages. For the first stage, we included four steps (Figure 1, flowchart). In the first step, we selected peer-reviewed papers published on the PubMed database, using the following keywords: deep learning AND diagnosis AND cancer (11,568 results). In the second step, the search was narrowed down to select clinical trials and randomized controlled trials to focus on deep learning application in the context of patient reported outcomes (116 results). In the third step, and to review the papers adequately, the search was restricted to papers with available free full-text (67 results). In the fourth search step, papers that were published in the last 3 years from 2021 to 2025 (53 results) were selected. In Stage 2, the suitability of the retrieved papers was assessed. In case the abstract did not present enough information that enables association with the work to the subject at hand, the full paper was reviewed to decide whether the article was appropriate for this study. Our exclusion criteria included: 1) papers that had been retracted (exclusion criteria 1), papers with content not matching the focus of the review (exclusion criteria 2), and papers that did not include model performance criteria (exclusion criteria 3). Ultimately, we ended up with 22 for the final analysis (Table 1). A list of the excluded papers with respective exclusion criteria is available in the Supplementary File 1.

## 4 Discussion

**FIGURE 2:**
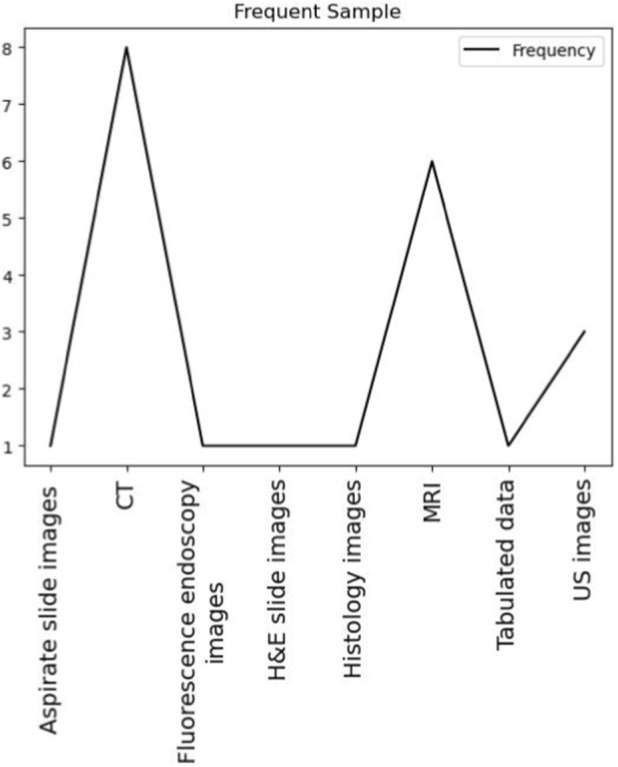
Frequency of sample type used for DL.

The conducted search identified 67 clinical trials and randomized controlled trials type of studies with full free texts available in the Pubmed database. Papers that were excluded from the study analysis are a total of 32: 3 papers due to retraction, 16 due to the content not matching the focus of the analysis conducted in this study, and 13 papers that did not include model performance criteria. The total number of papers that were filtered for further analysis is 22. Figure 1 shows a flowchart of the literature search. The analysis performed is divided into two stages. The first stage analyzes the type of deep learning (DL) models, sample data, and the performance metrics for each model used by type of cancer. The second stage studies the added values and limitations of the DL models used. To perform the first part of our analysis, 22 were filtered (Yin et al., 2022; Zheng et al., 2024; Ma et al., 2023; Yu et al., 2021; Meng et al., 2022; Zhan et al., 2024; Jayachandran Preetha et al., 2021; Fang et al., 2024; Wu et al., 2022; Liu et al., 2023; Chen et al., 2022; Pellicer-Valero et al., 2022; Zhang et al., 2024; Johnsson et al., 2022; Fremond et al., 2023; Wen-Zhi et al., 2022; Chen et al., 2023; Zhang et al., 2021; Kann et al., 2023; Zhang et al., 2022; He et al., 2016; Arabyarmohammadi et al., 2022). The basis of the filtration is to compile the studies that have the following variables available: Cancer Type, Sample Type, Number of Patients, Type of DL Model, Accuracy, Precision, Sensitivity, Specificity. The values of these variables were collected from the papers and tabulated in an Excel Sheet file (Supplementary File 2). Figure 2 presents the frequency of the Sample Type that was used for DL. It is shown that computed tomography (CT) scan images and magnetic resonance imaging (MRI) images are the most frequently used among the 22 papers with a total of 8 and 6 studies, respectively, using this type of sample data for the deployed DL models. Third to CT and MRI images is the ultrasound (US) images with a total of 3 studies. The least frequent type of samples, with only one paper using such types are: tabulated data including patient data, demographic information, and laboratory results, aspirate slide images, fluorescence endoscopy images, histology images, and hematoxylin and eosin (H&E) images. Table 1 shows the types of cancer disease that were studied in the filtered papers along with the size of the dataset (number of patients) included.

**FIGURE 3:**
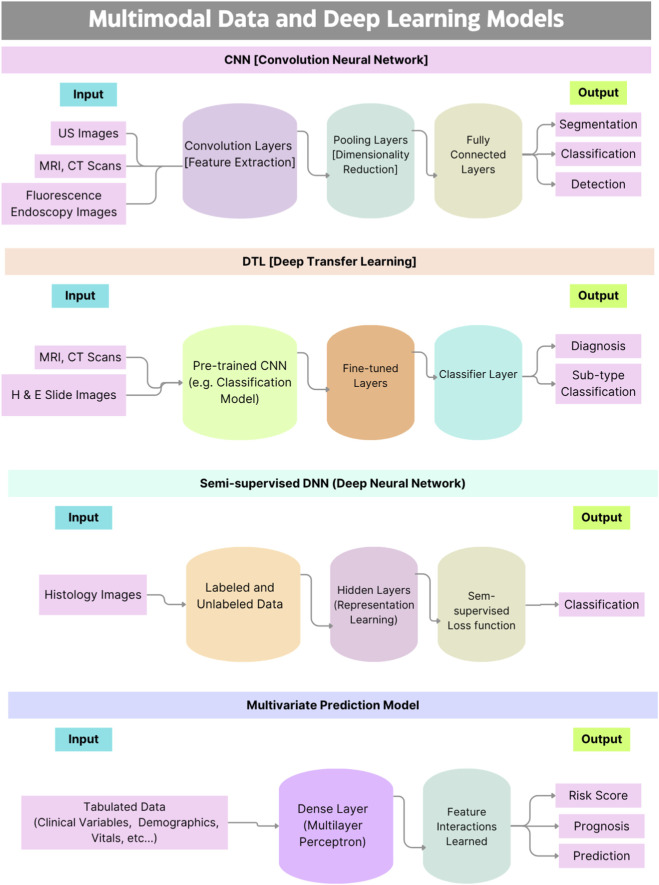
Flowchart of DL models and data types.

It is evident from the above table that some studies on specific cancer types have a limited number of samples from patients. This will be a factor in analyzing the potential of DL models in providing targeted diagnosis and treatment predictions for patients. Among the 22 studies, the following are the types of DL models that were deployed with its frequency across the selected papers: Deep Learning Network (1 study), 3D Convolutional Neural Network (CNN) (1 study), CNN (10 studies), Deep Transfer Learning (DTL) CNN (2 studies), DTL Classification Model (3 studies), High Resolution (HR) Network (1 study), Multivariate Prediction Model (1 study), Region-based Network (RCNN) (1 study), Semi-supervised Deep Neural Network (DNN) (1 study), aPROMISE Software (1 study). Figure 3 illustrates reviewed DL models and types of input data (Figure 3).

**FIGURE 4:**
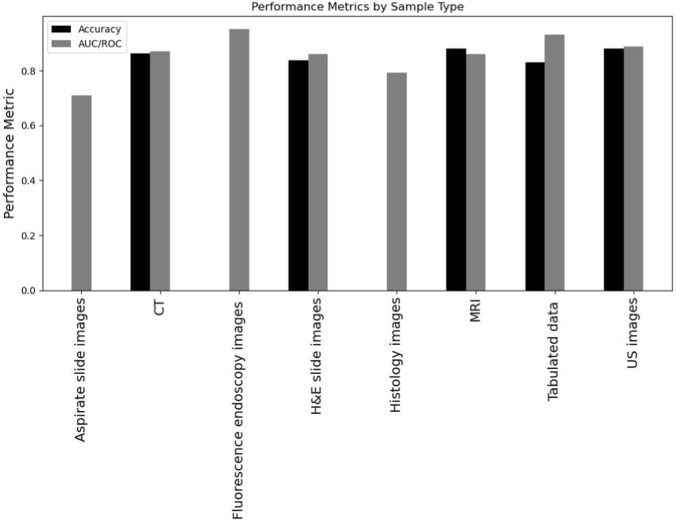
Performance of DL models in terms of accuracy and AUC/ROC metrics by sample type.

To study the performance of the models across the different studies, we chose to select the following metric parameters and observe the performance of the mentioned models across them: Accuracy and Area Under the Curve (AUC)/Receiver Operating Characteristic (ROC) - AUC/ROC. These two metrics were the most reported among the studies. Accuracy is an important performance metric as it measures how well the model can predict an outcome based on input data. As for AUC/ROC, it is a crucial metric to evaluate how well the model can distinguish between classes. It is worth mentioning that AUC/ROC analysis helps overcome subjectivity that is associated with relying solely on single sensitivity or specificity as indicators of accuracy. These metrics are often influenced by chosen diagnostic threshold for determining positivity, which is often randomly selected (Lin et al., 2025). Figure 4 presents the performance of the DL models in terms of the chosen performance metrics by sample type. It is evident that DL models using tabulated data may have higher AUC/ROC compared to models utilizing imaging data (CT scan and MRI). This is likely because tabulated data is more associated with molecular changes that are sometimes hidden and might not show up in CT or MRI images. As a result, it is recommended to implement DL with tabulated data and imaging data that are associated with molecular markers, specifically in situations where early diagnosis is crucial. This is also reflected in the samples using fluorescent microscopy imaging detecting specific proteins which also showed higher AUC/ROC. A table summarizing the performance metrics for the models is available in the Supplementary File 2.

**FIGURE 5:**
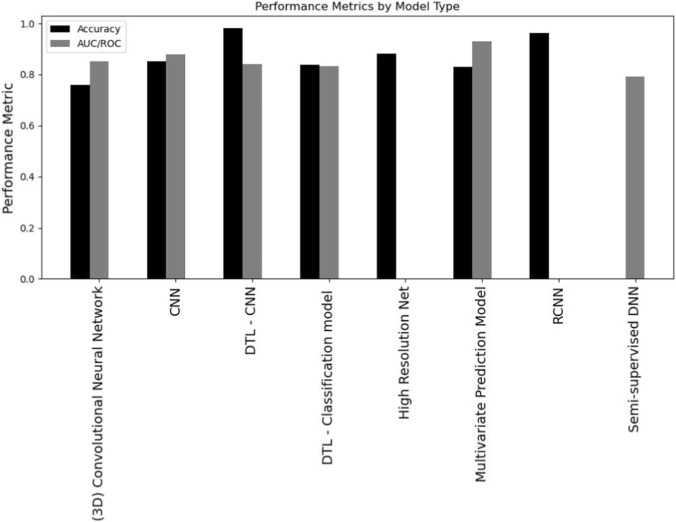
Performance of DL models in terms of accuracy and AUC/ROC metrics by model type.

Figure 5 presents the performance of the DL models in terms of the chosen performance metrics by algorithm type. From the results we can see that all deep learning algorithms have high accuracy.

For DL models implemented in studies on breast cancer, a common limitation across two studies was the small dataset size selected from a single center (Zheng et al., 2024; Meng et al., 2022). In addition to the fact that medical image datasets that are available for training are very limited due to privacy and confidentiality purposes (Meng et al., 2022). As reported in the studies, this might pose a selection bias and larger datasets from several centers should be considered in the future. Another limitation that may impact the performance of DL models in diagnosing or predicting breast cancer is the limited image resolution, especially for MRI-based CNN models. Therefore, studies suggest the combination of medical history, breast ultrasound or mammography, in addition to the MRI images used for diagnosis (Meng et al., 2022).

In the context of lung cancer (Zhang et al., 2022), the selected study presented two main limitations for implementing DL models. Like the limitation of breast cancer studies mentioned earlier, the dataset was obtained from a single center. In addition, models were not externally validated. External validation is important as it will prove that the performance of models remains consistent throughout different acquisition protocols and patient populations. Another limitation that was posed is that the selected study was retrospectively carried out. This makes the dataset prone to selection bias, and the researchers have limited control over the data collection process.

The study that analyzed ultrasound images for thyroid cancer is also a retrospective study (Zhan et al., 2024). The findings of the study will have to be further validated before any clinical application. In addition, data was obtained from a single healthcare facility, whereas, it is recommended to have data collection from multiple healthcare facilities to validate the applicability of results. These limitations were also observed in the studies that implemented DL models for prognosis and prediction of liver cancer (Liu et al., 2023; Chen et al., 2022). Another limitation is the analysis of only ultrasound images. Such images might not capture the dynamic malignancy risk characteristics, therefore, affecting the prediction accuracy of the DL model. It is recommended to conduct further development and improve prediction accuracy by having the ability to analyze dynamic images instead of only static.

A similar limitation of retrospective study with absence of further validation was also presented in the selected studies analyzing the brain tumor assessment response using MRI images (Jayachandran Preetha et al., 2021). An additional limitation is in MRI data being captured using various scanners and acquisition protocols suggesting variable performance of similar models. Standardization of acquisition protocols should be implemented to enhance the development of more precise predictive models.

Similarly, in the context of prostate, renal and pancreatic cancer, the limitations include small sample size and acquisition of dataset from a single center. In addition, the studies were retrospective demanding further external validation. In the case of prostate cancer, the model of a selected study was trained successfully on two datasets, but it still behaves differently on each of them (Pellicer-Valero et al., 2022). This highlights the importance of external validation with independent datasets from different sources.

In this study, we showcase and evaluate the potential of deep learning impowered models in cancer diagnosis and treatment through critical analysis of recent studies, however, we would like to acknowledge some limitations to this study. The first limitation is restricting the search date to the last 4 years. Our decision was driven by two reasons: First, the included period presented the latest advancements in artificial intelligence and machine learning (Dam et al., 2025). Second, our attempt to provide additional technical details on the included studies. Another limitation is that our analysis did not include the variability in the hyperparameters that were chosen in the different deep learning models. A third limitation is limiting the paper to the studies with available full-text to ensure appropriate review of included studies.

## 5 Emerging DL techniques and clinical implications

The immense potential for DL-powered workflows is currently attributed to several factors. Firstly, rapid advancement in infrastructure-level, including high-performance computing power and cloud computing (Sriraman et al., 2024; Hasimi et al., 2024). Secondly, the advancement in imaging techniques including functional imaging techniques, such as Magnetic Resonance Spectroscopy (MRS) and Contrast-Enhanced Harmonic Imaging Endoscopic Ultrasound (CEH EUS) (Jin et al., 2025). Thirdly, the emerging advanced pathology technologies such as Endoscopic Ultrasound-Guided Fine Needle Biopsy (EUS-FNB) (Jin et al., 2025). Fourthly, multimodal deep learning models are gaining increased attention due to the ability of these algorithms to process big volumes of structured and unstructured data across the full continuum of patient care (Yang et al., 2024). When utilizing DL algorithms for cancer prediction, diagnosis or treatment, it is crucial for clinicians to be aware that multimodal data improves the performance of models in prediction of recurrence, rather than relying on imaging alone (Mehri-Kakavand et al., 2025). Furthermore, and to avoid overfitting with respect to local imaging or practice protocols, external validation should be highly considered by clinicians for models that were developed in single centers or using retrospective datasets. In cases where DL models were developed with datasets that comprise of limited population, this external validation becomes even more necessary (Hira et al., 2023). DL and ML powered models can be effective tools for precision oncology at various stages. These models can analyze vast amount of diverse patient data to provide accurate and fast predictions. Secondly, DL algorithms discover new patterns that can be useful for personalized care management and treatment. Given its great and rapidly advancing potential, DL can improve early detection and the accurate diagnosis of cancer, however, it should be considered as a tool to support and not replace clinicians’ critical judgment and ethical decision making (Kotoulas et al., 2025).

## 6 Conclusion

AI and machine learning have been key to advancing healthcare. Deep learning is a branch of machine learning that imitates the human brain in utilizing neural networks to learn from complex data patterns and features, draw intelligent connections, and make informed decisions. The reassessment of clinical trials and randomized controlled trials utilizing deep learning frameworks demonstrated good performance of DL models in terms of accuracy and AUC/ROC metrics in various cancer types. Furthermore, our analysis also showed that implementing DL tools in analysis of data containing medical images with tabulated clinical data can result in good predictive performance of molecular cancer subtypes as in the case of breast cancer. This can be of important implications, specifically in treatment strategy and early diagnosis. Among the potential benefits of using deep learning tools in cancer management is the effective implementation of supervised and unsupervised machine learning strategies to integrate medical imaging and tumor multi-omics profiles to identify novel subtypes and prognostic markers per cancer type and subtype. This will help tailor therapies and guide clinical decisions to minimize side effects. Deep learning algorithms can include extraction of clinical information from electronic health records (EHRs) supporting personalized clinical decisions. Adapting deep learning tools to model complex relationships existing in unstructured data and multi-modal data provides potential to include data using non- or minimally invasive approaches that allow efficient and timely collection of physiological data. Another important aspect for using multi-modal deep learning empowered approaches is improving interpretability of the prediction models. This becomes crucial in ensuring trust and confidence in AI-supported human clinical decisions. Overall, through this analysis of clinical trials and randomized controlled trials, we support that the implementation of DL tools improves diagnosis and prognosis of various cancer types.

## Supplementary material

Supplementary FILE 1 List of papers excluded from this systematic review.

Supplementary FILE 2 Characteristics of filtered studies included in this systematic review.
